# Psychometric Properties of the Hebrew Translation of the Patient Activation Measure (PAM-13)

**DOI:** 10.1371/journal.pone.0113391

**Published:** 2014-11-20

**Authors:** Racheli Magnezi, Saralee Glasser

**Affiliations:** 1 Department of Management, Bar Ilan University, Ramat Gan, Israel; 2 Gertner Institute for Epidemiology and Health Policy Research, Sheba Medical Center, Tel Hashomer, Ramat Gan, Israel; University Of São Paulo, Brazil

## Abstract

**Objective:**

“Patient activation” reflects involvement in managing ones health. This cross-sectional study assessed the psychometric properties of the Hebrew translation (PAM-H) of the PAM-13.

**Methods:**

A nationally representative sample of 203 Hebrew-speaking Israeli adults answered the PAM-H, PHQ-9 depression scale, SF-12, and Self-efficacy Scale via telephone.

**Results:**

Mean PAM-H scores were 70.7±15.4. Rasch analysis indicated that the PAM-H is a good measure of activation. There were no differences in PAM-H scores based on gender, age or education. Subjects with chronic disease scored lower than those without. Scores correlated with the Self-efficacy Scale (0.47), Total SF-12 (0.39) and PHQ-9 (−0.35, P<0.0001), indicating concurrent validity. Discriminant validity was reflected by a significant difference in the mean PAM-H score of those who scored below 10 (72.1±14.8) on the PHQ-9 (not depressed) compared to those scoring ≥10 (i.e. probable depression) (59.2±15.8; *t* 3.75; P = 0.001).

**Conclusion:**

The PAM-H psychometric properties indicate its usefulness with the Hebrew-speaking Israeli population.

**Practice Implications:**

PAM-H can be useful for assessing programs aimed at effecting changes in patient compliance, health behaviors, etc. Researchers in Israel should use a single translation of the PAM-13 so that findings can be compared, increasing understanding of patient activation.

## Introduction

“Patient activation” is a term used to describe the extent to which individuals understand that they must play an active role in managing their own health and healthcare, and the extent to which they feel able to fulfill that role. Hibbard and her colleagues conceptualized patient activation as encompassing a range of elements important for self-management that extend beyond any single health behavior [1]. As a measure of this concept, they developed the Patient Activation Measure and its short form (PAM-13) [1], [2]. This is a 4-level model of health behavior, in which the lowest level indicates that the patient may not believe his/her role in self-care management is important. The second level indicates that the patient lacks confidence or knowledge to take an active role. The third level indicates that the patient is beginning to be active and at the highest level, patients actively attempt (even with difficulty) to maintain healthcare management behaviors over time [3].

The PAM-13 and its underlying conceptualization can help healthcare providers understand patients' strengths or weaknesses in optimizing their healthcare and health status. It has been associated with improved patient compliance and health outcomes [4]–[7]. Studies in several countries have used the PAM-13 for a variety of populations, including the elderly [8], [9], mental health patients [10] and those with specific diseases [11]–[13].

The PAM-13 has been translated and validated in several languages including Dutch [14], Danish [15], Spanish [16], and German [17]. A Hebrew translation has been used in studies of primary care patients [18] and participants in a social health network for persons with chronic disease [19]. However, the psychometric properties of the Hebrew translation have not been heretofore assessed.

The current study assessed the psychometric properties of the Hebrew translation (PAM-H) of the PAM-13. It was hypothesized that the PAM-H would: (a) have good internal consistency and that Rasch analysis would confirm its value as a measure of activation; (b) correlate positively with measures of well-being and self-efficacy; and (c) correlate negatively with depressive symptoms.

## Methods

### Participants

This cross-sectional study included a nationally representative sample of 203 Hebrew-speaking Israeli adults 25 to 80 years of age, with no exclusion criteria.

### Instruments

#### Patient Activation Measure (PAM -13)

The PAM-13 is a self-report questionnaire with 13 items. Raw scores range from 13-52. These are converted to “activation scores” ranging from 0–100 [20], which can be analyzed as a continuous variable. Higher scores indicate increasing patient involvement.

### Comparison Measures

To consider concurrent validity of the PAM-H the following instruments were used:

#### Patient Health Questionnaire-9 (PHQ-9)

The PHQ-9 is a self-report questionnaire for screening for symptoms of depression based on the Diagnostic and Statistical Manual of Mental Disorders-IV. It has a sensitivity of 93% and specificity of 85% in primary care settings [21]. Each of the nine items is scored 0 to 3, resulting in a total score ranging from 0 to 27. Higher scores indicate more severe symptoms. A cut-off score of ≥10 is considered to indicate at least a mild depressive episode [22]. The Hebrew translation of the PHQ-9 has also been validated in a primary care setting [23].

#### Short Form-12 Health Survey (SF-12)

The SF-12 is a 12-item questionnaire that measures an individual's subjective sense of well-being. Scores range from 0 to 100, with higher scores indicating better quality of life [24]. The SF-12 is composed of eight subscales, as well as physical and mental health composite scales (PCS and MCS, respectively). The Hebrew translation of the SF-12 has been validated [25].

#### Self-efficacy Scale

Self-efficacy is an individual's belief in his/her ability to conduct specific activities. Measures of self-efficacy have been shown to predict behavior [26], [27]. The Self-efficacy Scale [28], [29] is a ten-item questionnaire that has been translated into Hebrew [30]. Each item is rated on a four-point scale that ranges from “not at all true” (1) to “exactly true” (4). Higher scores indicate a greater sense of self-efficacy. Internal consistency of the Hebrew version has been reported as 0.89 [31].

### Translation

In the current study, the PAM-13 was translated as recommended by the World Health Organization for cross-cultural adaptation of self-report measures [32]. The translation from English to Hebrew and back-translation from Hebrew to English were done by professionals familiar with the terminology of the field, knowledgeable in both English- and Hebrew-speaking cultures. A bilingual Expert Panel (including the translators and other professionals) was convened to identify and resolve expressions or concepts that were open to misunderstanding or confusion. Discrepancies were discussed, consensus was achieved and the translation and its cultural appropriateness were confirmed. The final version was pretested on fifteen individuals, followed by debriefing, and found to be clear and acceptable.

### Procedure

The sample design and data collection were conducted by the Cohen Institute for Public Opinion Research at Tel Aviv University. A probabilistic stratified sampling of households was built based on the official statistical areas classified according to socio-demographic characteristics. Areas were then matched with the computerized list of subscribers to the national telephone company and households randomly chosen. The proportion of Israeli households that possess landline telephones is 85%.

Potential participants were contacted by telephone. If the person who answered met study criteria (Hebrew-speaker, aged 25 to 80), the purpose of the research, expected length of interview and nature of participation (responding to questionnaire items and providing basic demographic data) were described. The person was then specifically asked if he/she was willing to participate and if verbal informed consent was granted, then the interviewer proceeded with the telephone interview. Only one adult in each household was interviewed. No personal identification (name, ID number) was requested or recorded. Participant consent was recorded by the interviewers and evidenced by proceeding with the interview upon receipt of consent. When consent was not received (in 153 cases), these were noted as ‘refusals’ and so reported. The interviews were conducted during December 2013.

### Sample Size

The sample size was determined using the Epi Info program, v.6 (CDC, 2011). Based on the results of an earlier Israeli study [18], a sample size of approximately 200 was needed to detect a change in PAM-13 levels vis-à-vis mean PHQ-9 or SF-12 scores. The calculation was based on a power of 1-β = 80% and a p-value of α = 0.05.

### Data Analysis

Data were entered onto an Excel worksheet and analyses were conducted using SAS Version 9.2 for SOLARIS software. The PHQ-9 scores were analyzed both as continuous and dichotomous variables. The PHQ-9 scores were dichotomized into 0-9 (not depressed) and ≥10 (probable depression), where indicated. Discrete variables were analyzed by chi-square test and continuous variables by Pearson Product Correlation or t-test for difference in means. Mean scores were compared by t-test, or by GLM procedure for variables with more than two categories. Psychometric analysis of the PAM-H was conducted using the Rasch measurement model with Winsteps software [33]. Infit and Outfit were computed and values between 0.5 and 1.5 were considered to represent a good fit. Ten subjects who answered all of the questions with “strongly agree” (i.e. lacking response variability) were not included in the Rasch analysis. Floor and ceiling effects were determined according to the recommendation that an effect is present if more than 15% of the participants achieve the lowest or highest possible score, respectively [34]. The construct validity of the PAM-H was analyzed as a continuous measure after raw scores (range 13–52) were converted to ‘activation scores’ (1–100) [20]. Only thirteen PAM-H questionnaires had missing items: nine with one missing item and the remainder each had 2–4 missing items. Scores of missing items were calculated as the mean score of the remaining items. Socio-demographic variables were categorized.

### Ethics

The study was approved by the Ethics Committee of Bar Ilan University. Receipt of written informed consent was not feasible in the context of this telephone-interview study based on a randomly-chosen sample of households. The researchers' description of requesting and receiving verbal informed consent (noted above) was described to the Ethics Committee on the application for approval, and approval was granted for verbal informed consent.

## Results

Among 395 individuals who were contacted, 203 (51.4%) participated in the study; 153 (38.7%) declined to be interviewed and 39 (9.9%) telephones were not answered after several attempts. It was not possible to distinguish the respondents from non-respondents because this was a random population telephone survey and non-participants could not be asked for personal information.

The participants were divided almost evenly between males and females, and between those with up to 12 years of education and those with more than 12 years (Table 1). Almost half of the participants were 55 years-of-age or older (44.5%) and 62.5% had an average income level. Regarding health characteristics, 22.4% of the participants reported having at least one chronic disease (e.g., high blood pressure, diabetes, asthma).

**Table 1:** Mean PAM scores by demographic and health characteristics.

Characteristic	Total N (%)^a^	PAM Score Mean (SD)	P-value
**Total**	203 (100.0)	70.7 (15.4)	
**Sex**			0.37
Male	99 (48.8)	71.7 (14.7)	
Female	104 (51.2)	69.7 (16.1)	
**Age Group**			0.86
25–34	24 (11.9)	71.6 (18.6)	
35–44	39 (19.3)	69.9 (12.6)	
45–54	49 (24.3)	72.6 (15.4)	
55–64	36 (17.8)	70.2 (17.4)	
65+	54 (26.7)	69.4 (14.7)	
**Education**			0.58
≤12 years	94 (46.3)	71.3 (16.1)	
>12 years	109 (53.7)	70.1 (14.8)	
**Income Level**			<0.001^b^
Far below average	26 (13.0)	60.6 (13.2)	
Around average	125 (62.5)	73.3 (14.3)	
Far above average	10 (5.0)	73.0 (11.7)	
Not disclosed	39 (19.5)	67.5 (16.1)	
**Health Status**			0.03
No Chronic Disease	156 (77.6)	71.9 (15.7)	
≥1 Chronic Disease	45 (22.4)	66.4 (13.8)	
**Regular Medication**			0.37
No	117 (61.9)	71.6 (16.3)	
Yes	72 (38.1)	69.6 (14.3)	

a Not including missing values.

b Significant for “Far below average” vs. “About average” comparison only, as self-described in comparison to the average Israeli family income level.

PAM-H scores ranged from 27.9 to 100, with a mean of 70.7±15.4 (median 73.1; mode 75.3). The Cronbach's alpha for internal consistency was 0.77. Rasch analysis indicated that the PAM-H is a good measure of activation [35]. All items had very good measurement properties and calibrated almost identically to the PAM-13. Infit values of the PAM-H ranged from 0.70 to 1.35 and outfit values ranged from 0.73 to 1.45 (Table 2). The item difficulty structure (not shown) corresponded very closely with a data file of 52,713 subjects composed of many diverse samples, indicating that the PAM-H produces the structure expected if it is measuring the activation construct.

**Table 2:** PAM-H Item Fit Statistics (N = 193)^a^.

PAM-H Item	Infit Value^b^	Outfit Value^c^
PAM-H 1	1.08	0.81
PAM-H 2	1.07	1.28
PAM-H 3	1.06	1.11
PAM-H 4	1.22	1.09
PAM-H 5	1.35	1.45
PAM-H 6	1.33	1.44
PAM-H 7	0.99	0.82
PAM-H 8	0.88	0.88
PAM-H 9	0.88	0.85
PAM-H 10	1.09	1.01
PAM-H 11	0.77	0.76
PAM-H 12	0.88	0.85
PAM-H 13	0.70	0.73

a Only participants with diversity in their answers were included.

b Infit reflects the similarity of observed responses from model expected response, being most sensitive when the item and respondent are close together on the activation scale.

c Outfit is reflects unexpected observations with respect to the respondent's other responses. It is most sensitive when the item location on the scale is far away from the person's location on the scale.

There were no differences in the mean PAM-H score based on gender, age group or education (Table 1). Those who described their family income level as ‘far below average’ had significantly lower PAM-H scores than did those with at least average income, or than the nearly one-fifth of participants who preferred not to disclose their income level (P = .0006). Participants who reported any chronic disease had significantly lower PAM-H mean scores than did those with no chronic disease (6.4±13.8 vs. 71.9±15.7, respectively; P = 0.03). There was no significant difference in mean PAM-H scores between those who did or did not take medication regularly. However, when the groups were analyzed according to a combined variable including both income level and chronic disease, it was found that those with any chronic disease whose income was far below average had significantly lower PAM-H mean scores (53.1±12.5) compared to those who were at the same income level, but had no chronic disease (64.6±17.0) or those with an average or above average income with (70.7±11.5) or without chronic disease (74.0±14.7; P = 0.0001). No floor or ceiling effects were found. The lowest score of 0 was not reported and only 4.9% of the participants achieved the maximum score of 100.

SF-12 scores ranged from 17–44 (35.6±6.0) and Self-efficacy Scale scores ranged from 13–40 (34.9±4.6). PHQ-9 scores ranged from 0–20 (3.8±4.2), with 10.4% of the participants scoring 10 or above, which indicates a probable depressive episode.

Concurrent validity was demonstrated by correlations in the expected direction with the three comparison questionnaires. As shown in Table 3, PAM-H scores were positively correlated with the Self-Efficacy Scale and SF-12 Total scores (0.47 and 0.39, respectively; P<0.0001) and negatively correlated with the PHQ-9 depression scale (−0.35, P<0.0001). PAM-H scores were also positively correlated with each of the SF-12 subscales, ranging from r = 0.38 for the General Health subscale to r = 0.22 for the Role-Physical and Role-Emotional scales, as well as for the Physical and Mental Health Composite scales.

**Table 3:** Correlation of PAM with PHQ-9, SF-12 and Self Efficacy Scales.

PAM Correlated with:	N	r_spearman_	P
Self Efficacy Scale	203	0.47	<0.0001
PHQ-9 (depression)	203	−0.35	<0.0001
SF-12 Total Score	202	0.39	<0.0001
**SF-12 Subscales**			
Bodily Pain	202	0.25	0.0003
Physical Functioning	201	0.26	0.0002
Role-Physical	202	0.22	0.001
General Health	203	0.38	<0.0001
Mental Health	201	0.28	<0.0001
Role-Emotional	201	0.22	0.0015
Social Functioning	201	0.24	0.0007
Vitality	203	0.32	<0.0001
Physical Health Composite	203	0.36	<0.0001
Mental Health Composite	203	0.34	<0.0001

Discriminant validity was indicated by the significant difference in the mean PAM-H score of those who scored below 10 on the PHQ-9 (not depressed) compared to those scoring ≥10 (probable depression); (72.1±14.8 vs. 59.2±15.8; *t* 3.75; P = 0.001).

## Discussion and Conclusion

### Discussion

This study assessed the psychometric properties of the Hebrew translation of the PAM-13. Rasch analysis indicated the unidimensionality of the PAM-H and that it works very well as a measure of activation, with all items having very good measurement properties and item difficulty calibrating almost identically to the PAM-13. The mean PAM-H score (70.7) was somewhat higher than that reported in the original study of Hibbard et al. (61.9) on the development of the PAM-13 [1]. This might be related to the availability and accessibility of healthcare in Israel compared to the United States. Israel's National Health Insurance Law ensures that every citizen is fully covered for basic services through one of four health maintenance organizations [36], all of which have widely distributed networks, including primary and secondary clinics for preventive care as well as for acute and chronic care, generally with long-term continuity of care. This accessibility may contribute to a general sense of familiarity and comfort with the healthcare system on the part of the public, leading to higher degrees of patient activation. In other countries overall mean PAM scores have also been found lower than in our Israeli sample. For example, the mean score for the German translation was 67.1 [17], and 61.3 in Dutch [14] and 64.2 in Danish [15] validation studies. Further investigation into this issue may be warranted, as the health systems vary from country to country. Indeed, Wong et al. [37] found that patient activation was related to various aspects of primary care, including accessibility and having a positive relationship with the primary care provider.

Within the study group, the mean PAM-H score was significantly lower among participants with chronic diseases compared to those without such conditions. In the present study, among those with chronic conditions, the mean PAM-H score was similar to that reported in other patient populations [10], [12], [13], [17]. This was also found by Rademakers et al. [14] in a Dutch population and by Maindal et al. [15] in a Danish population, who also reported lower mean scores related to poorer self-reported health. The only demographic factor significantly related to PAM-H scores was having far below average income.

As expected, PAM-H scores were positively correlated with a subjective sense of well-being and self-efficacy, and negatively correlated with depressive symptoms. Although these correlations were not extremely high, they were all significant and similar to those found in other studies comparing the PAM-13 with scales measuring these constructs. For example, Hibbard et al. [1], reporting on the development of the original PAM, found a correlation of r = 0.38 with the SF-8, and Green et al. [10], validating use of the PAM-13 with mental health patients reported a correlation of r = 0.30 with self-reported quality-of-life. Studying patients with multiple sclerosis, Stempleman et al. [13] reported a correlation of r = 0.42 of the MS Quality-of-Life Scale with the PAM-13. Correlations for the sub-scales of the SF-36 were also similar, ranging from r = 0.21 to r = 0.37 [10], while in the present study the correlations for the parallel SF-12 scales ranged from r = 0.22 to r = 0.38. The PAM-H correlation with the Self-efficacy Scale was higher, similar to that found by Stempleman et al. [13], although to a lesser degree than that found in a study [12] of patients undergoing elective lumbar spine surgery. Negative correlations of the PAM-H with depressive symptoms were similar to those found in other studies [10], [13].

An earlier study of 278 patients visiting a primary health care clinic in southern Israel employed a Hebrew translation of the PAM-13 [18], which was similar to that used following the cross-cultural translation procedure employed in this validation study. The mean PAM-13 score, as well as the correlations with the total SF-12 and PHQ-9 scores, were identical with those found here, implying the robustness of these findings among the Israeli population.

Conducting this study by means of a telephone interview could be considered a limitation of the present research. However, it should be noted that in our earlier study [18], there was no significant difference in PAM-13 scores for the various age groups or between those who were interviewed face-to-face or by telephone. Furthermore, Pinto-Meza et al. [38] investigated this issue and found that telephone interviews were reliable for use with the PHQ-9.

### Conclusions

The Hebrew translation of the PAM-13 has been found reliable and valid for use with the Hebrew-speaking Israeli population.

### Practice Implications

An important aspect of the PAM-13 is its usefulness in assessing intervention programs aimed at effecting changes in measures such as patient compliance, health behaviors [4]–[7], and even depressive symptoms [18]. It is recommended that further research with the PAM-H document its value for this purpose among additional populations and patient sub-groups. It is further recommended that in a multi-cultural country such as Israel, similar translation-validation studies be conducted on large minority groups, such as the Arab and Russian populations. It is also important that researchers in Israel use a single translation of the PAM-13 so that findings can be compared, increasing our understanding of patient activation.
